# Epidermal Growth Factor Receptor Gene in Non-Small-Cell Lung Cancer: The Importance of Promoter Polymorphism Investigation

**DOI:** 10.1155/2018/6192187

**Published:** 2018-10-14

**Authors:** Vladimir Jurišić, Jasmina Obradovic, Sonja Pavlović, Nataša Djordjevic

**Affiliations:** ^1^Faculty of Medical Sciences, University of Kragujevac, Svetozara Markovica 69, 34000 Kragujevac, Serbia; ^2^Institute of Biology and Ecology, Faculty of Science, University of Kragujevac, Radoja Domanovica 12, 34000 Kragujevac, Serbia; ^3^Institute of Molecular Genetics and Genetic Engineering, University of Belgrade, Vojvode Stepe 444a, 11010 Belgrade, Serbia

## Abstract

Recently, epidermal growth factor receptor (EGFR) was a key molecule in investigation of lung cancer, and it was a target for a new therapeutic strategy, based on molecular analyses. In this review, we have summarized some issues considering the role of EGFR in lung cancer, its coding gene, and its promoter gene polymorphisms (SNPs) -216G/T and -191C/A in non-small-cell lung cancer (NSCLC). The position of the SNPs indicates their significant role in EGFR regulation. The accumulation of knowledge regarding SNPs lately suggests their significant and important role in the onset of carcinogenesis, the prediction of the onset of metastases, the response to therapy with TKI inhibitors, and the onset of toxic effects of the applied therapy. Based on this, we suggest further studies of the relationship of clinical significance to SNPs in patients with lung tumors.

## 1. Non-Small-Cell Lung Cancer

Over the years, many scientific reports referred to lung cancer as “the leading cause of death” worldwide [1–6]. Non-small-cell lung cancer (NSCLC) is the most common form of lung cancer and accounts for about 85% of all cases of cancer [7–10]. Classical chemotherapy has been a major option for this type of tumor for many years, but the mortality remained high. For this incurable disease, the hope seems to lie in preventive medicine, i.e., various education strategies about risk factors, introduction of new programs for early cancer screening and early diagnostics, and providing equal chances for proper treatment to all patients [6, 8].

Carcinogenesis is a multistep process that usually takes many years to develop, as there are several mechanisms that prevent it, including the immune system, antioxidative system, and DNA repair mechanisms [11, 12]. The recent development of new techniques and methods has increased the knowledge of molecular mechanisms during carcinogenesis [13–15]. These mechanisms, including increased gene amplification and protein expression, abnormal cell activation, allelic disbalance, and epigenetic mechanisms [13–20], might be just the top of the iceberg for all undiscovered interactions and signaling networks that are present in cancer cells. Studies in animal transgenic mice have shown that during carcinogenesis, one of the important molecules is epidermal growth factor receptor (EGFR) [13, 20].

## 2. Epidermal Growth Factor Receptor

Epidermal growth factor receptor (EGFR), usually being overexpressed in many cancers, such as non-small-cell lung cancer and colorectal and breast cancers [21], has drawn scientists' attention early. It is a transmembrane protein with the N-terminal extracellular-ligand binding domain, transmembrane lipophilic domain, and C-terminal intracellular tyrosine kinase (TK) domain. The binding of ligand to the extracellular domain leads to formation of homo- or heterodimers within the EGFR family and a subsequent activation of the TK domain. In normal cells, it is a trigger molecule for many important processes, including growth, development, and differentiation. In altered cells, it conducts many abnormal messages through a signaling network cascade, leading to carcinogenesis [22]. Binding of the adaptor proteins such as Grb2 and Shc induces activation of three main signaling pathways Ras/MAPK, PI3K/Akt, and JAK/STAT, which in altered cells lead to uncontrolled proliferation, angiogenesis, inhibition of apoptosis, invasion, metastasis, and immortalization [13, 23, 24]. These key molecules of signaling cascades might also be affected by gene mutations, altering the process of carcinogenesis [13, 20, 25, 26].

In nontransformed cells, EGFR activation triggers inhibitory mechanisms including dephosphorylation and inactivation with inducible feedback inhibitors, acting as tumor suppressors [27]. There are three main mechanisms that lead to EGFR activation in malignant cells: increased EGFR expression, increased ligand production, and the presence of EGFR-activating mutations [21]. In NSCLC with overexpressed EGFR, the inhibition of the receptor signaling has been introduced as a targeted treatment, with tyrosine kinase inhibitors (TKIs), such as gefitinib and erlotinib, rendered optimal in carriers of *EGFR*-activating mutations [21, 28, 29].

## 3. *EGFR* Gene Regulation

*EGFR* is located at the short arm of the chromosome 7 (7p11.2), spans about 200 kb, contains 28 exons, and encodes a protein of 1210 amino acids [30]. Currently, the regulation of *EGFR* expression is not completely understood, and different factors have been proposed to have a role in the process. Namely, most of the eukaryotes have regulatory elements for binding transcription factors (so-called “TATA” and “CAAT” sequences), located about 30–80 bp upstream of the start transcription site [31, 32]. *EGFRs'* 5′ region differs from the 5′ region of the most of eukaryotes, as it has less regulatory elements and high GC content in the promoter region, providing multiple start sites for the initiation of RNA transcription [31, 32].

*EGFR* promoter activation requires transcription factor Sp1, for which multiple binding sites were discovered [31, 33–37]. *EGFR* transcription is upregulated by at least three enhancers that act cooperatively: two of them localized upstream, i.e., near the start transcription site, and the third one in introne [38–40]. In the context of *EGFR* regulation, different *cis* and *trans* elements are reviewed, including TP53 (so-called “guardian of the genome”), p63, epidermal growth factor (EGF) responsive DNA-binding protein 1 (ERDBP-1), early growth response factor 1 (Egr-1), EGFR-specific transcription factor (ETF) (ETR–EGFR), cis-acting EGF receptor transcriptional repressor, repressor regulatory element in the first introne of *EGFR*, transforming growth factor *β* (TGF-*β*), GC-binding factor (GCF), microsatellite CA sequence, AP1, and AP2 [33, 34, 41–50].

## 4. *EGFR* Gene Amplification and Overexpression in Tumors

Expression of EGFR is a complex process, and it differs in normal and cancerous cells. Although the genetic mechanism of EGFR protein overproduction is not completely elucidated, it represents a very common event in different tumors [21] and is usually associated with a more progressive stage of disease, worse prognosis, and higher mortality [51, 52]. In the literature, there is a certain controversy concerning the correlation among *EGFR* gene amplification, EGFR overexpression, and the efficacy of the TKI treatment. Namely, while earlier investigations did not observe clear relationship between EGFR expression and clinical outcomes for the NSCLC patients treated with TKI [53, 54], succeeding studies reported significant association of both high *EGFR* gene copy number (due to gene amplification or chromosome polysomy) and high protein expression with better response to gefitinib or erlotinib [55–57].

Some studies showed no correlation between *EGFR* gene amplification and protein expression [58, 59], while others reported the association [60–62]. It was observed that the amplification of *EGFR*, as a result of gene rearrangement in chromosome 7, leads to formation of aberrant RNA [60]. Several studies showed that *EGFR* amplification, as well as *EGFR*-activating mutations, are associated with the increased iRNA expression and in turn with a better therapy outcome [55, 63, 64]. Described inconsistency in reports suggests that *EGFR* genetic variations might play a role in both NSCLC carcinogenesis and TKI therapy success.

## 5. *EGFR* Variations

The most common *EGFR* somatic mutations are positioned in the TK domain, i.e., within exons 18 to 24 [30, 65]. These mutations are clustered around the EGFR ATP-binding pocket, affecting ATP affinity and altering sensitivity to TKIs [65]. Most of them, including E746_A750del and L858R, are classified as activating or “gain-of-function” mutations and could be found in NSCLC patients that respond well to gefitinib or erlotinib [66]. Others, such as T790M, usually emerge later during the treatment, causing secondary resistance to TKI therapy [67]. Currently, both are considered pharmacogenetic biomarkers in oncology, which could help in predicting the outcome of the treatment [68–70]. Yet, even with the *EGFR* somatic mutation data, a part of the observed interindividual difference in clinical response to gefitinib and erlotinib remains unexplained.

**Figure 1:**
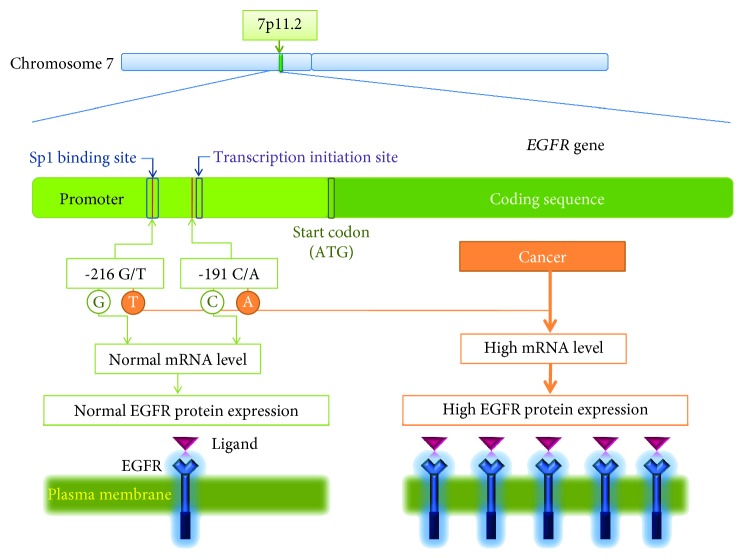
*EGFR* gene location on chromosome 7 and functional characteristics of two SNPs -191C/A and -216G/T placed in the *EGFR* promoter region.

There are numerous germline single nucleotide polymorpshisms (SNPs) found within EGFR [71], some already associated with increased risk of certain tumors [72–74] or with altered response to drug therapy [15, 19, 75–78]. Among the best studied EGFR SNPs are -216G/T and -191C/A, whose location within the *EGFR* promoter region indicates their potential role in EGFR regulation. Namely, -216G/T (rs712829) is placed within the transcription factor Sp1 binding site of the *EGFR* promoter, and -191C/A (rs712830) 4bp upstream from one of the start transcription binding site [32–34, 40, 75] (Figure 1). A low level of linkage disequilibrium (LD) that was observed between -216G/T and other important *EGFR* SNPs suggests its independent role in gene regulation, with G to T substitution resulting in significant increase of both promoter activity and mRNA expression [40, 79]. On the other hand, tight LD with other variations and lower effect on EGFR activity have been described for -191C/A [40, 70].

## 6. Ethnicity and Variants of EGFR

Ever since the significance of EGFR variations for the clinical response to therapy of lung cancer has been recognized [63, 64, 80], they have been the subject of intense research around the world. Based on the obtained results, modern classification and diagnostics of the lung cancer are nowadays performed based on molecular analysis [81].

It has been observed that *EGFR* variants occur more frequently in Asia, unlike KRAS mutations, which are more common in Caucasians [82–84]. This suggests that there are significant interethnic differences in the molecular basis of carcinogenesis of the lung cancer. In addition, it was shown that the frequency of EGFR mutations is also higher in women, nonsmokers, and patients with adenocarcinoma, as compared to other types of lung cancer [85]. In our study from 2016, for the first time in our knowledge, the white people in the Balkans have described the frequency -216G/T and -191C/A, we found that the distribution of these SNPs coincides with their distribution in the whites from other areas [85]. Another investigation, which was carried out in a Caucasian population from the Balkan country, also showed the correlation of EGFR polymorphisms with the histological type of cancer, with the variant alleles being the most frequent in adenocarcinoma [86].

On the other hand, the interethnic differences in incidence, mortality, prognosis, and survival of NSCLC are already known [87–89]. In most cases, these differences can be associated with a different frequency of *EGFR* variations [40, 88–94]. Although many polymorphisms and mutations of *EGFR* have been described, the two polymorphisms of the promoter region, namely, -216G/T and -191C/A, were shown to be especially important [40], as they convey ethnicity-dependent genetic susceptibility for lung cancer [40, 90–94]. The frequencies of EGFR variations in different ethnic populations are summarized in Table 1.

Based on the previous reports, there are interethnic differences in frequency distribution of *EGFR* promoter SNPs. Namely, in Caucasians and Afro-Americans, -216G/T is much more frequent than in Asians [40, 94] while -191C/A was detected almost only in Caucasians [40] and with extremely low frequency in East Asians [94–99].

Furthermore, these polymorphisms have been associated with the localization of tumor metastases. Namely, as the process of cell proliferation and differentiation is strictly related to EGFR, tumor metastasis should be affected by variations in *EGFR*. In line with the expectations, significant differences in genotype and allele frequencies of the -216G/T polymorphism between the patient group with the pleural metastasis in comparison with the nonmetastasis group have been observed [100]. Based on these findings, the authors have concluded that the polymorphism in exon 13 of the EGFR gene might be one of the molecular mechanisms of pleural metastasis of lung cancer.

Having in mind the importance of these polymorphisms for the NSCLC therapy outcome, both ethnic background and the cancer stage should be considered in making a decision on a proper treatment approach.

## 7. TKI and EGFR Variants

Tyrosine kinase inhibitors (TKIs) specifically bind to the intracellular tyrosine kinase (TK) domain of the EGFR receptor and thereby prevent the transmission of a signal directed to the development of malignancy. In the NSCLC treatment, first-generation TKIs include gefitinib and erlotinib, the second generation TKI involves afatinib and dacomitinib, and the third generation involves recently approved osimertinib [101–106].

There are many reasons for obtaining resistance to drugs used in targeted therapy. In regard to TKIs, one of the possibilities includes EGFR wild-type allele amplification, highlighting the importance of EFGR genotype for the treatment efficacy [107].

Regarding safety, according to earlier *in vitro* data, neither -216G/T nor -191C/A seems to be associated with cytotoxicity of different TKIs, including erlotinib [79]. These findings have been supported by few *in vivo* reports, where correlation between any of the two *EGFR* promoter polymorphisms and the occurrence of skin rash or diarrhea with gefitinib treatment was not detected [108, 109]. However, numerous other studies involving advanced NSCLC patients on gefitinib therapy demonstrated the opposite. Namely, a higher response rate and prolonged progression-free and overall survivals but also significantly higher risk of treatment-related rash and diarrhea were observed in carriers of at least one -216T allele [15, 110]. In similar studies on the role of -216G/T and -191C/A polymorphisms in patients with advanced NSCLC treated with gefitinib or erlotinib, variant haplotypes were associated with the clinical benefit, time to progression, and the overall survival [19, 109] but also with the gastrointestinal and skin drug toxicities [17, 111]. The similar association found between -216G/T variant allele and the successful clinical response to anti-EGFR monoclonal antibodies such as cetuximab or panitumumab further supports the proposed role of *EGFR* promoter polymorphism in therapy targeting NSCLC patients [112].

## 8. Conclusion

EGFR is usually overexpressed in many epithelial cancers; thus, the inhibition of its signaling pathway has been introduced as a potential very successful treatment of NSCLS. Yet, there are pronounced interindividual differences in response to TKIs, with EGFR being among the most important determinants. Although *EGFR* gene amplification, gene mutation, and chromosome polysomy have all been associated with TKI therapy success, there is a part of the observed interindividual difference in clinical response to therapy that remained unexplained. *EGFR* SNPs -216G/T and -191C/A discussed here are located in the promoter region of the gene, which indicates their potential role in EGFR regulation. The data from *in vivo* studies involving NSCLC patients demonstrate that both these SNPs but especially -216G/T affect efficacy and safety of the TKI treatment, suggesting their importance in making a decision on a proper therapy approach.

## Figures and Tables

**Table 1:** EGFR -191C/A and -216G/T minor allele frequencies in lung cancer patients of different ethnicities.

	Minor allele frequency			
SNP	Caucasians	Asians	African Americans	Publication
rs712830 (-191C/A)	0.136 (6/44)	0.000 (0/46)	0.000 (0/48)	Liu et al. [40]
	0.114 (37/324)	NA	NA	Cusatis et al. [95]
	NA	0.000 (0/54)	NA	Choi et al. [90]
	0.071 (13/184^∗^)	NA	NA	Liu et al. [15]
	0.099 (19/192)	NA	NA	Giovannetti et al. [17]
	0.128 (85/662)	NA	NA	Winther Larsen et al. [78]
	0.226 (19/84)	NA	NA	Obradović et al. [85]
	NA	0.035 (9/260)	NA	Bashir et al. [96]

rs712829 (-216G/T)	0.318 (14/44)	0.071 (3/46)	0.292 (14/48)	Liu et al. [40]
	0.444 (144/324)	NA	NA	Cusatis et al. [95]
	NA	0.040 (2/54)	NA	Choi et al. [90]
	0.400 (73/184^∗^)	NA	NA	Liu et al. [15]
	0.440 (144/328)	NA	NA	Gregorc et al. [109]
	0.401 (77/192)	NA	NA	Giovannetti et al. [17]
	NA	0.020 (23/1128)	NA	Dong et al. [97]
	NA	0.050 (14/282)	NA	Liu et al. [98]
	NA	0.056 (8/142)	NA	Jung et al. [19]
	NA	0.283 (361/1276)	NA	Guo et al. [99]
	0.326 (216/662)	NA	NA	Winther Larsen et al. [78]
	NA	0.130 (60/460)	NA	Zhang et al. [110]
	0.310 (26/84)	NA	NA	Obradović et al. [85]
	NA	0.287 (491/856)	NA	Guo et al. [100]
	NA	0.596 (155/260)	NA	Bashir et al. [96]

NA: not available; ^∗^ population mainly Caucasian.
